# Natural biomaterials in the management of the aortic valve pathology. Biomedical and clinical aspects: A review

**DOI:** 10.17305/bb.2024.11009

**Published:** 2024-09-24

**Authors:** Igor Mokryk, Illia Nechai, Christoph Schmitz, Ihor Stetsyuk, Oleksandr Talalaiev, Borys Todurov

**Affiliations:** 1 Department of Adult Cardiac Surgery, Heart Institute, 02660, Kyiv, Ukraine; 2 Auto Tissue Berlin GmbH, Berlin, Germany

**Keywords:** Biomaterials, pericardium, aortic diseases, neocuspidization, decellularization, structural valve deterioration, glutaraldehyde, aortic valve neocuspidization

## Abstract

Heart valve diseases are a prevalent cardiovascular pathology worldwide, affecting nearly 2.5% of the population. Degenerative aortic stenosis is the most common form of heart valve disease. The treatment options include surgical or transcatheter procedures. There are two main categories of valve prostheses available: mechanical heart valves constructed from synthetic materials and bioprosthetic heart valves made from natural biomaterials. The choice of valve type depends on various factors, including the underlying medical condition, suitability for anticoagulation, valve durability, and the patient’s age and preferences. Mechanical heart valves have the advantage of long-term durability. However, patients receiving mechanical implants are subjected to lifelong anticoagulation therapy with an increased risk of thromboembolism and bleeding. Natural biomaterials do not require long-term anticoagulation. However, they experience degenerative changes leading to structural valve deterioration that may require reoperation. The purpose of this article is to review the role of natural biological materials used for aortic valve replacement or repair, assess their biomedical and clinical advantages and limitations, and analyze the direction and perspectives of future development.

## Introduction

A biomaterial is a material designed to interact with biological systems for medical applications. These materials should be compatible with blood and capable of interacting with the physiological environment of cells or tissues when implanted. Biomaterials can be classified into two primary categories: synthetic and natural. General requirements for cardiac valve biomaterials include biocompatibility, high mechanical stability, resistance to degradation and calcification, reproducibility, and cost effectiveness. Synthetic materials encompass traditional materials, such as metals, polymers, and ceramics. These materials offer certain advantages over natural biomaterials. Their properties, such as 3-dimensional structure, mechanical strength, and degradation time, can be precisely controlled during synthesis [1]. However, synthetic materials also have limitations, primarily concerning biocompatibility, which can lead to issues, such as inflammation, thrombosis, or thromboembolism [2]. Patients with mechanical valves must take anticoagulants for life, facing an increased risk of thromboembolism and hemorrhage. The incidence of significant bleeding in these patients ranges from 0.34 to 2.91 per 100 patient-years [3–5], while the rate of thromboembolic complications is 2.80 per 100 person-years [6]. Natural biomaterials are derived from native tissues of autogenic (same individual), allogenic (same-species donor), or xenogenic (animal) sources (Table 1). Autologous tissue is currently the best option due to its superior functionality and non-immunogenicity [7]. Xenogeneic tissues are harvested from animal sources, such as cows, pigs, or horses. Natural biomaterials typically outperform synthetic ones in terms of biocompatibility, plasticity, and low thrombogenicity. However, their primary drawbacks are degeneration and calcification, which lead to structural valve deterioration (SVD). The most significant factors contributing to SVD are the host’s immune response to antigenic epitopes and alterations caused by treatment and sterilization processes [8, 9]. Improving current tissue treatment and sterilization methods, enhancing decellularization protocols, and using autologous and xenogeneic tissues from genetically modified animals may help mitigate SVD.

**Table 1:** Classification of biological materials used in surgical aortic valve replacement

**Classification**	**Source**	**Examples**	**Usage in aortic valve replacement**
Autologous materials	Patient’s own tissues	- Autologous pericardium - Autologous pulmonary valve (ross procedure)	- Used for neocuspidization (ozaki procedure) - Pulmonary valve replacement (ross)
Allogenic materials	Human donor (cadaveric)	- Allograft (homograft) valve	- Used in cadaveric valve replacements, often for patients with valve infections
Xenogenic materials	Animal sources	- Xenogenic pericardium (bovine, porcine, equine) - Xenogenic valve (porcine valve)	- Bovine/porcine pericardium and valves are commonly used in bioprosthetic valves
Synthetic hybrid materials	Processed biological material	- Decellularized xenogenic or allogenic tissue - Tissue-engineered heart valves (experimental)	- Decellularized tissues reduce immunogenic response, experimental tissue engineering

### Autogenic materials

The use of human autologous pericardium for reconstructing the aortic valve (AV) dates back to the late 1960s [10, 11]. The first documented case of AV cusp extension using pericardium occurred in 1963 when Ross treated aortic regurgitation by enlarging a single cusp with fresh autologous pericardium [12]. In 1964, Björk and Hultquist presented pathological findings from two cases where AV cusp extensions were performed, likely with fresh autologous pericardium. One patient died five months later, showing thickened and calcified pericardium. The other patient required reoperation due to severe valvular regurgitation within 3.5 months of the procedure and died during the surgery [10]. In 1969, Edwards described a surgical technique for total valve replacement using autogenous tissue, tested in vitro. He performed the procedure on two patients, one of whom died one week post-surgery. An autopsy revealed that the reconstructed AV was functioning [13]. Bahnson et al. reported two single-leaflet replacements and three triple-leaflet extensions using autologous pericardium in 1970. Though all patients had residual regurgitation, three were doing well at follow-ups conducted 1.5 and 4.5 years post-operation [11]. These early attempts to use fresh autologous pericardium for AV reconstruction yielded unsatisfactory results due to tissue thickening and shrinkage, necessitating reoperations despite good initial hemodynamic outcomes [10, 11, 13]. To address tissue retraction, Love and colleagues proposed immersing the pericardium in a 0.6% glutaraldehyde (GA) solution [14]. This method significantly improved outcomes compared to using fresh pericardium. GA fixation promotes cross-linking of the remaining free amino groups, reducing biodegradation, preserving anatomical structure, increasing collagen fiber stiffness and durability, and making the tissue biocompatible and non-thrombogenic [8, 15, 16]. The use of GA-treated autologous pericardium (GTAP) became particularly significant when complete AV reconstruction techniques using autopericardium were introduced into clinical practice. Duran et al. [17] initially developed the concept of using GTAP for AV reconstruction, which was further refined by Ozaki et al. [18]. Duran et al. introduced an innovative method for total AV replacement using GTAP in 1995, reporting excellent immediate results. A decade later, Al Halees et al. published a long-term outcome analysis of the procedure, involving 92 patients divided into two groups. Group I included 27 patients who received AV reconstruction with bovine pericardium, and Group II consisted of 65 patients whose AV was reconstructed with GTAP. The mean age in the general cohort was 30 years, and the mean follow-up was 10.5 ± 4 years. Freedom from SVD was 78 ± 1% at ten years and 55 ± 10% at 16 years for Group I, compared to 80 ± 5% at ten years and 58 ± 9% at 15 years for Group II. The mean time to valve degeneration was 8.8 years (±3.6 years) and was similar for both groups [19]. This study demonstrated the acceptable long-term outcomes and excellent hemodynamic performance of AVs reconstructed with GA-treated pericardial tissue. It also showed that both bovine and autologous GA-treated pericardium can be safely used for AVR. In another study, Chan et al. reported long-term outcomes of AV reconstruction using GTAP in 11 patients, with a mean follow-up of 6.5 years. Freedom from SVD, thromboembolism, and calcification was 100%, while freedom from infective endocarditis (IE) was 72.7% and from reoperation 63.6%. The study indicated that AVR with GTAP offers excellent durability up to 7.5 years, with no calcification [20]. The largest AV reconstruction experience with GTAP was reported by Ozaki et al. [18]. In 2007, Ozaki performed his first aortic valve neocuspidization (AVNeo) using autologous pericardium. This procedure involves treating the patient’s pericardium with a 0.6% GA solution for 10 min, followed by three washings in saline for 6 min each. Yamashita et al. compared the tensile strength and elasticity of GTAP with non-calcified, calcified, and decalcified aortic leaflets. Their findings showed that the mechanical strength of GTAP is four times higher than that of non-calcified aortic leaflets [21]. In 2018, Ozaki reported outcomes for 850 consecutive patients who underwent AVNeo with GTAP. The median patient age was 71 years (range 13–90 years), and the mean follow-up was 53.7 ± 28.2 months, with the longest observation period being 118 months. Eight years after surgery, the average peak pressure gradient on the AV was 15.2 ± 6.3 mmHg. Actuarial survival was 85.9%, the reoperation rate was 4.2%, and moderate or greater recurrent aortic regurgitation occurred in 7.3% of patients [22]. These results demonstrated the feasibility and favorable mid-term outcomes of AVNeo with GTAP. Since the initial reports, AVNeo’s popularity has grown worldwide. Recent studies have added to the body of literature on the clinical outcomes of the AVNeo procedure and the properties of GTAP. Gardin et al. [23] compared the morphological characteristics and cell viability of pericardial tissue used in the AVNeo procedure to native tissue. They found that the extracellular matrix (ECM) organization in GA-treated pericardium was altered, which reduced cell viability compared to native tissue. However, GA-treated pericardium did not show cytotoxic effects on murine fibroblasts, nor did it inhibit endothelial cell repopulation [23]. Pirola et al. analyzed the outcomes of 71 patients who underwent AVNeo with GTAP, with a median follow-up of 20.7 months. No in-hospital deaths occurred, and freedom from major adverse valve-related events was 97%. Four patients (5.6%) had mild/moderate aortic regurgitation, but none had severe regurgitation, and no reoperations were necessary [24].

Krane et al. reported similar outcomes in their study of 103 patients post-Ozaki procedure, with 97.0% freedom from moderate or greater aortic regurgitation during a mean follow-up of 426 ± 270 days and 96.1% overall freedom from reoperation [25]. Mylonas et al. conducted a meta-analysis of 22 studies involving 1,891 patients who underwent the AVNeo procedure. The patient cohort had a mean age of 43.2 ± 24.5 years. The peak gradient was 15.7 ± 7.4 mmHg, and the incidence of moderate aortic regurgitation was 0.25% at the latest follow-up. Freedom from reoperation was 98.0% at one year, 97.0% at three years, and 96.5% at five years. However, endocarditis remains a concern, accounting for over half of the reoperations, with a risk of 0.5% per patient-year [26]. Dilawar et al. reviewed 12 studies involving 1427 patients who underwent GTAP-based AVR between 2009 and 2020. The mean patient age was 64.95 years, and 52.1% were male. Most patients had preoperative aortic stenosis (75.4%), followed by aortic regurgitation (19.62%) and a combination of both (3.64%). Of the 1,427 patients, 25 (1.75%) died, 3 (0.21%) had thromboembolic events, 13 (0.91%) developed IE, and 16 (1.12%) required reoperations. Endocarditis was the primary cause of reoperation in 69% of cases. All studies reported a reduction in postoperative peak pressure gradient [27]. Benedetto et al. analyzed outcomes from UK centers that performed the Ozaki procedure and conducted a meta-analysis comparing AVNeo outcomes with other biological AV prostheses. They concluded that the mid-term risk of valve-related events was comparable between AVNeo and other biological AV substitutes [28]. The plasticity of GTAP and xenopericardium allows for the development of novel surgical techniques tailored to individual anatomy. Modifying the standard AVNeo technique using preoperative CT measurements of the aortic root enables personalized AV leaflets to be prepared before bypass, reducing ischemic and bypass times [29, 30]. Prosthetic valve endocarditis (PVE) remains a serious complication in AV surgery. However, GTAP has demonstrated resistance to infection. In Mourad et al.’s study of 52 patients, five reoperations were required during a mean follow-up of 11.2 ± 4.8 months, all due to PVE. In four of these cases, tissue-engineered bovine pericardium was used for valve reconstruction [31]. Other studies report comparable incidences of PVE between AVNeo and standard aortic valve replacement (SAVR) [26, 28]. Todurov et al. reported higher GTAP resistance to infection in their experience using AVNeo principles for pulmonary valve correction in adults. Pulmonary valve neocuspidization (PVNeo) was performed on three patients. Two were intravenous drug users who also underwent tricuspid valve replacement with a biological prosthesis. Follow-up showed excellent outcomes in two cases, with no signs of recurrent infection. The third patient was readmitted nine months later due to endocarditis related to continued drug abuse. Vegetations were found on the bioprosthesis, but the PVNeo valve remained intact with good hemodynamic performance [32]. AVR using GTAP is considered a safe and effective procedure, offering favorable short- to mid-term outcomes, including significant hemodynamic improvements and low reoperation rates. However, further studies with longer follow-up periods are needed to better assess the long-term benefits of this procedure compared to mechanical and bioprosthetic valves [27, 28].

**Table 2:** Fixation methods for biological materials used in surgical aortic valve replacement

**Fixation method**	**Mechanism**	**Materials used**	**Advantages**	**Disadvantages**
Glutaraldehyde fixation	Cross-links collagen and proteins, stabilizing the tissue	Xenogenic pericardium (bovine, porcine, equine) Xenogenic valve (porcine valve)	- Prevents immune rejection - Preserves structural integrity	- Risk of calcification- Limited long-term durability
Formaldehyde fixation	Cross-links proteins by forming methylene bridges	Rarely used in modern valves due to toxicity	- Long-term tissue preservation	- Toxicity - Risk of structural weakening
Epoxy fixation (e.g., epoxy compounds)	Cross-links collagen fibers and proteins, stabilizing tissue	Xenogenic pericardium Xenogenic valves	- Reduces calcification- Provides durable cross-links	- Complex and expensive - Limited long-term data
Polyethylene glycol (PEG)	Polymer coating that prevents tissue dehydration and calcification	Xenogenic pericardium	- Prevents calcification - Non-toxic	- Short-term stability in some studies - Experimental use
EDC/NHS (carbodiimide) fixation	Cross-links proteins without aldehyde toxicity	Xenogenic pericardium Experimental applications	- Low toxicity - Reduces calcification	- Limited clinical data- Technical complexity
Genipin fixation	Natural cross-linking agent derived from plants	Experimental on Xenogenic tissues	- Low toxicity - More biocompatible than glutaraldehyde	- Limited clinical data- Expensive and experimental
Decellularization (chemical process)	Removes cellular material, leaving collagen scaffold	Allogenic valve Xenogenic valve	- Reduces immunogenic response - Preserves structure	- May weaken tissue - Potential loss of mechanical properties

### Allogenic materials

Homografts play a vital role in addressing heart valve pathology. Sourced from human heart donors or autopsy material, they are commonly stored in nitrogen vapor or an antibiotic solution. Homografts are primarily used in pediatric cardiac surgery, with the Ross procedure being one of the most popular methods for correcting AV pathology. This technique involves replacing the malformed aortic root with a pulmonary autograft, while continuity between the right ventricle and pulmonary artery is restored with a homograft [33]. The immunogenicity of homograft heart valves depends on the preservation method used, which affects their cellular viability [34]. Cellular viability is linked to the immune response due to the presence of human leukocyte antigen (HLA) class I and II antigens. Homografts with full cellular viability present these antigens on all valve endothelial cells, triggering an immune response. In contrast, completely decellularized homografts have no cellular viability and do not present HLA class I and II antigens, resulting in a lower immune response [34]. Vogt et al. studied the impact of blood group incompatibility on outcomes of AVR with homografts. They found no statistically significant difference in overall event-free survival related to blood group incompatibility. However, they recommended histological and immunohistochemical assays for confirmation [35]. In adult cardiac surgery, homografts have shown promise in treating IE of the AV. Galeone et al. examined the outcomes of cryopreserved aortic homograft implantation in 104 patients, most of whom had AVR or aortic root replacement (ARR) due to IE. IE complications included annular abscesses in 82% of cases, mitral valve endocarditis in 14%, and tricuspid valve endocarditis in 14%. Hospital deaths occurred in 12% of patients, and the mean survival time was 13.9 ± 1.2 years. During follow-up, 39 patients (42%) died, and 25 (26%) required late reoperation, primarily due to aortic homograft degeneration (18%) or homograft-related endocarditis (6.7%) [36]. These findings suggest that the long-term reoperation rates for homografts are comparable to other biological prostheses. Since aortic valve IE is often complicated by annular abscesses, ARR with a homograft may sometimes be the only viable option.

### Xenogeneic materials

The first successful xenograft valve implantation in humans was performed in 1965 [37]. However, early xenopericardial valve replacements had a high failure rate—nearly 60% within a year—mainly due to the host’s acute immune response and subsequent calcification, leading to valvular stenosis or regurgitation [38, 39]. Various measures, including electrolysis washing, sodium periodate treatment, and GA fixation, have been employed to increase the resistance of xenograft valves to calcification and improve their biocompatibility. While these approaches enhance the functionality and mechanical strength of xenografts, SVD) remains a significant issue [39]. SVD is age-dependent and more prevalent in younger patients due to their more robust immune system response to epitopes on xenogeneic tissue [40, 41]. Young patients also experience accelerated calcium metabolism, contributing to higher rates of SVD in bioprosthetic heart valves (BPHVs). Furthermore, toxic residues from the chemical treatment of xenografts can damage cells, initiating calcification and SVD [42]. Various calcification-preventive agents, including sodium dodecyl sulfate (SDS), α-amino oleic acid, and ethanol with Tween-80, have been tested, but none have fully resolved the issue. Xenopericardium, particularly from bovine and equine sources, provides an abundant tissue resource for bioprosthesis construction [43]. Studies have shown that BPHVs made from tissue derived from different animal sources demonstrate similar hemodynamic performance, with no significant difference in calcification rates between bovine and porcine BPHVs [44, 45]. GA treatment remains the standard for enhancing the durability and biocompatibility of these valves, which have been successfully used in clinical practice. Table 2 describes common chemical fixation methods used for biological materials in AVR procedures and their mechanisms and advantages. The development of stented BPHVs in the 1970s involved sewing GA-fixed bovine pericardium onto a flexible stent, allowing synchronous opening of the leaflets [46]. However, early versions experienced SVD due to leaflet tearing. Over time, thinner and more flexible stents were introduced, reducing valvular stress and allowing larger valves to be implanted. Despite these advances, stented BPHVs are associated with patient–prosthesis mismatch (PPM), where the valve size is too small for the patient’s native valve [47]. This issue led to the development of stentless BPHVs, which allow for more natural valve opening and reduce the incidence of PPM [48]. Modern commercial BPHVs come in four types: stented, stentless, sutureless, and percutaneous [49]. Their leaflets are primarily made from bovine, equine, or porcine pericardium [50]. Surgical aortic valve replacement (SAVR) is a standardized procedure with good long-term outcomes. Valve choice should be tailored to the patient’s risk profile, anatomical factors, and personal preferences. Petersen et al. examined long-term outcomes in 354 non-elderly adults undergoing biological AVR, finding that 6.3% had grade 2 or greater aortic regurgitation after a mean follow-up of 78.7 ± 38.1 months [51]. Another study by Danial et al. reported that freedom from SVD at ten years was 73.3%, while freedom from moderate SVD was 50.3%. The rate of freedom from major adverse valve-related events at ten years was 69.7% [52].

**Table 3:** Comparative table of biological materials used in surgical aortic valve replacement

**Material**	**Main source**	**Main chemical fixation methods (if required)**	**Pros**	**Cons**
Autologous pericardium	Patient’s own pericardium	Glutaraldehyde fixation Fresh	- Excellent biocompatibility - Avoids immune rejection - No need for anticoagulation - Flexible and pliable	- Need to replace pericardium - Technical complexity - Longer ischemic time - Long-term data scarce
Autologous valve	Patient’s own pulmonary valve (Ross procedure)	Not required	- Excellent biocompatibility - Avoids immune rejection - No need for anticoagulation - Durable, especially in young patients - Grows with the patient	- Complex surgery - Potential failure of pulmonary valve replacement - Higher reoperation rates in adults due to pulmonary autograft dilation
Allogenic valve	Cadaveric valve (homograft)	Cryopreservation antibiotic sterilization	- No need for anticoagulation - Less immune rejection (no α-gal epitops) - Good for infected aortic valves - Possibility to replace aortic root	- Limited availability - Calcification risk - Durability lower compared to mechanical valves
Xenogenic pericardium	Bovine, porcine, equine	Glutaraldehyde fixation	- Good biocompatibility - Readily available - Mild immunogenic response	- Higher risk of calcification over time - Limited long-term durability - SVD development
Xenogenic valve	Porcine valve	Glutaraldehyde fixation	- Good biocompatibility - Less anticoagulation needed compared to mechanical valves - Wide availability	- Risk of structural degeneration and calcification - Durability lower than mechanical valves

The field of transcatheter aortic valve implantation (TAVI) has advanced as an alternative approach to open-heart surgery, particularly for high-risk patients. Prof. Cribier performed the first percutaneous valve implantation in humans in 2002, marking a significant milestone in the development of this technique [53]. In the NOTION trial, which randomized lower-risk patients with severe aortic stenosis to receive either TAVI or SAVR, there were no significant differences in major clinical outcomes. Severe SVD occurred in 1.5% of TAVI patients and 10.0% of SAVR patients, indicating that TAVI patients experienced a lower incidence of severe SVD. However, the overall risk of bioprosthetic valve failure was comparable between the two groups [54]. Neocuspidization of the AV may also be performed using xenopericardium. One of the most significant experiences using xenopericardium for AV reconstruction was reported by Song et al. This study included 262 consecutive patients who received AV reconstruction with bovine pericardium. The mean follow-up duration was 36.0 ± 17.1 months, and all patients completed follow-up. The study reported no in-hospital mortality, suggesting the procedure’s safety. However, three late deaths (1.1%) occurred during the follow-up period, all attributed to non-cardiac causes. Seven patients (2.7%) required reoperation, primarily due to endocarditis (five cases) and disruption of sutured leaflets (two cases). AV regurgitation was absent or trivial in 87.3% of patients, indicating successful valve function. Mild regurgitation was observed in 11.2% of patients, mild to moderate regurgitation in 1.2%, and moderate to severe regurgitation in 0.4%. The mean valve gradient and valve orifice index were reported as 10.6 ± 5.3 mmHg and 1.3 ± 0.4 cm^2^/m^2^, respectively. The study concluded that xenopericardial valves for AV replacement offer positive outcomes in terms of mortality, reoperation rates, valve competence, and hemodynamic performance [55]. Mitrev et al. compared the outcomes of total AVNeo using xenopericardium (xAVNeo) vs bioprosthetic valve replacement in patients with severe aortic stenosis and a small aortic root. The study included 412 patients between 2003 and 2018, with 114 patients receiving xAVNeo and 298 receiving AVR. After propensity matching, the final cohort consisted of 222 patients. The study’s primary endpoints were early mortality, 6-year mortality, and freedom from reoperation. The mean follow-up was 3.4 ± 3.1 years, with a 95% follow-up completion rate. Early mortality rates were 8.1% for the AVR group and 9.9% for the xAVNeo group, with no statistically significant difference between the two. Six-year survival probabilities were comparable—89.9% for the AVR cohort and 88.8% for the xAVNeo cohort. However, the study revealed that xAVNeo using bovine pericardium was associated with a higher SVD rate than AVR. Neocusps in the xAVNeo group showed significant degeneration, leading to an increase in the mean gradient from 6.1 ± 2.3 to 22.7 ± 11.5 mmHg. Cusp sclerosis was the most common reason for reoperation in the xAVNeo group, which had a higher reoperation rate (1.92% vs 0.26% per patient-year in the AVR group). Freedom from reoperation at six years was 84.8% for xAVNeo, compared to 100% in the AVR group. Mitrev et al. concluded that while early clinical outcomes and 6-year survival rates were similar, xAVNeo with bovine pericardium was associated with a higher rate of SVD and lower freedom from reoperation compared to SVR. These findings suggest that xAVNeo, despite initial clinical benefits, requires further refinement to improve long-term durability [56]. While autologous and allogenic materials offer the advantage of natural tissue integration, xenogenic materials provide a practical solution for patients requiring bioprosthetic valves. Each material involves tradeoffs regarding durability, immune response, and reoperation risks, which must be carefully considered based on the patient’s age, health condition, and long-term prognosis (Table 3). Advancements in tissue engineering and chemical fixation methods continue to push the boundaries, promising better outcomes and more durable solutions for future generations of AVR patients.

### Future perspectives

Reducing the immunogenicity of natural biomaterials has been shown to enhance clinical outcomes [57]. Decellularization, a process that removes cellular components (e.g., DNA and RNA) from the ECM while preserving its mechanical integrity, is one promising approach [58]. This method minimizes host immune responses and promotes the remodeling of the implanted tissue by the patient’s own cells [59]. Decellularized tissues offer advantages such as regenerative potential, favorable functional properties, and biocompatibility [60]. Decellularization is a promising alternative to GA fixation because it reduces residual DNA, cell fragments, and membranes, thereby decreasing immunogenicity [61, 62]. The remodeling potential of decellularized scaffolds is particularly advantageous in pediatric applications, where future growth is essential [63]. Decellularization techniques involve chemical, biological, and physical methods. Chemical detergents such as Triton X-100, sodium deoxycholate, and sodium dodecyl sulfate are often used, along with biological enzymatic agents like DNase, RNase, and Trypsin. Physical methods, such as mechanical agitation or pressure, can further improve decellularization effectiveness [64–67]. Decellularized pericardial patches have been used in pediatric cardiac surgery to correct atrioventricular (AV) pathology, although the outcomes have often been suboptimal due to the early onset of SVD [68–71]. However, these challenges may be attributed more to the complex anatomical conditions than to the patches themselves. On the other hand, the use of decellularized patches in unicuspid AV repair has shown promising results, demonstrating their potential effectiveness in these procedures [72–74]. While these results are encouraging, more data on the application of decellularized patches in adult patients is needed to assess long-term efficacy and safety. In summary, while decellularized materials show potential for AVR, further research and clinical trials are necessary to evaluate their efficacy and safety compared to traditional valve replacement options. Sterility is crucial for medical implants, including those made from biological tissue. Several sterilization methods are used, such as chemical agents (e.g., peracetic acid and ethylene oxide), UV radiation, and gamma irradiation. However, gamma irradiation can significantly alter the structure and biomechanics of pericardial tissue [75]. Supercritical carbon dioxide is being investigated as a promising alternative sterilization method for biomaterials [76]. Another factor affecting the durability of bioprosthetic heart valves is the expression of various antigenic epitopes, including α-Gal, in xenograft valves. While α-Gal epitopes are absent in humans, they are present in non-primate mammals. The anti-Gal antibody, which comprises about 1% of immunoglobulins in humans, can trigger an immune response, leading to early rejection of xenograft BPHVs [77]. Therefore, it is critical to inactivate or mask these epitopes during BPHV preparation [78]. Naso et al. conducted a quantitative evaluation of α-Gal epitopes on seven GA-treated, commercially available BPHVs. They found that only one out of the seven valves completely masked α-Gal epitopes [79]. Based on these results, the authors recommended implementing α-Gal ELISA testing as a quality control measure for commercially available BPHVs. Recently, genetically modified animals with reduced levels of α-Gal epitopes have garnered growing interest [80, 81]. For example, Rahmani et al. [82] introduced a novel GA-fixed porcine pericardial BPHV from transgenic pigs, which demonstrated excellent durability in vitro.

## Conclusion

Natural biomaterials play a crucial role in the surgical management of aortic valve pathology, offering biocompatibility, flexibility, infection resistance, low thrombogenicity, and the non-immunogenicity of autologous tissue. However, tissue degradation, typically occurring within ten years, remains a challenge. Continued research into developing new biomaterials, improving sterilization methods, and refining implantation techniques is essential for improving long-term outcomes in AVR.
